# H2A.X N-terminal acetylation is a newly identified NAA40-mediated modification that is responsive to UV irradiation

**DOI:** 10.1186/s13072-025-00608-3

**Published:** 2025-07-16

**Authors:** Ariel Klavaris, Costas Koufaris, Roberta Noberini, Maria Kouma, Christina Demetriadou, Alessandro Ghiringhelli, Nikolas Dietis, Tiziana Bonaldi, Antonis Kirmizis

**Affiliations:** 1 Department of Biological Sciences, University of Cyprus, 2109 Nicosia, Cyprus; 2 Cyprus Cancer Research Institute, 2109 Nicosia, Cyprus; 3 Department of Experimental Oncology, IEO, European Institute of Oncology IRCCS, 20139 Milan, Italy; 4 Department of Oncology and Haematology-Oncology, University of Milano, Via Festa del Perdono 7, 20122 Milan, Italy; 5 Experimental Pharmacology Laboratory, Medical School, University of Cyprus, Nicosia, Cyprus

**Keywords:** NAA40, H2A.X, Acetylation, Chromatin, Epigenetics, Histone, Variant, DNA damage

## Abstract

**Background:**

N-terminal acetylation (Nt-Ac), mediated by N-terminal acetyltransferases (NATs) is one of the most abundant protein modifications occurring approximately in 80% of all eukaryotic proteins. In contrast to the broad spectrum NATs, the human N-alpha-acetyltransferase 40 (NAA40) is highly specific, currently known to Nt-acetylate only the two histone proteins H4 and H2A, which share an Ser(1)-Gly(2)-Arg(3)-Gly(4) N-terminal sequence. Previous work from our lab and others has highlighted the biological and clinical relevance of this NAA40-mediated modification.

**Results:**

In this study, by performing in silico analysis of protein sequences combined with biochemical assays we identify the histone variants H2A.X and H2A.J and the chromatin remodeler SMARCD2 as new potential substrates of human NAA40. Subsequently, focusing on H2A.X, we show for the first time by mass spectrometry analysis that H2A.X is N-terminally acetylated (Nt-acH2A.X) within human cells. Next, we demonstrate that NAA40 specifically interacts and N-terminally acetylates histone H2A.X, in vitro and within cells. Finally, we provide evidence that H2A.X N-terminal acetylation is responsive to Ultraviolet B (UVB)-induced DNA damage and its associated enzyme NAA40 affects the survival of cells exposed to UVB irradiation.

**Conclusion:**

Our findings identify H2A.X as a novel *bona fide* substrate of NAA40. Moreover, the responsiveness of H2A.X N-terminal acetylation to UV-induced DNA damage indicates that this is a dynamic modification with potential biological functions.

**Supplementary Information:**

The online version contains supplementary material available at 10.1186/s13072-025-00608-3.

## Background

One of the most abundant eukaryotic protein modifications is N-terminal acetylation (Nt-Ac), occurring on an estimated 80% of proteins. This modification is deposited either co- or post-translationally by an evolutionarily conserved family of enzymes, collectively known as N-terminal acetyltransferases (NATs) [1]. Historically, the first identified NAT complexes were defined by a promiscuous substrate selectivity, consistent with the prominence of this modification across the eukaryotic proteome. The first exception to this broad substrate spectrum NATs was the identification of the highly selective NAA40 [2, 3]. Specifically, human NAA40 was shown to selectively acetylate the N-terminal tip on histones H4 (Nt-acH4) and H2A (Nt-acH2A), which both harbor the N-terminal Ser(1)-Gly(2)-Arg(3)-Gly(4) recognition sequence [4]. Interestingly, a recent study showed that mutations in any of the four amino acids negatively affect the catalytic efficiency of NAA40 towards these mutants, further confirming its specificity towards the SGRG motif [5].

Importantly, our lab and others have demonstrated that loss of NAA40 can affect vital biological processes [6]. In yeast, deletion of the NAA40 homolog was shown to impact ribosomal-DNA silencing and the response to caloric restriction via altered Nt-acH4 [7, 8]. In human cells antagonistic crosstalk of H2A/H4 Nt-Ac with the phosphorylation of the same N-terminal serine residue (H4S1ph) was linked to lung cancer cell invasion and resistance to chemotherapy in colorectal cancer cells [9, 10]. Nevertheless, whether NAA40 targets other proteins beyond H2A and H4 remains currently unknown. Given the recent emergence of NAA40 as a potential novel therapeutic target [11], it is important to define more comprehensively the NAA40 substrate repertoire, in order to assist the characterization of its biological functions and potential side-effects of therapeutic targeting.

Beyond the canonical core histones—H2A, H2B, H3 and H4—eukaryotic genomes also encode variants that display distinct expression profiles and biological functions [12]. To date, several variants have been identified for histones H2A and H3 including H2A.X, H2A.Z and macroH2A for the former, and H3.1, H3.2 and H3.3 for the latter. In contrast to H2A and H3, only a limited number of variants have been identified for histones H2B, and a single H4 variant has been recently identified known as H4G [13–17]. Histone variants are subjected to various modifications similar to canonical histones that can influence their functional properties [18–20]. Whether histone variants are acetylated on the N-terminal amino group of their first amino acid residue remains elusive.

In this study, we initially performed in silico analysis and identified histone variants H2A.X and H2A.J and the chromatin remodeler SMARCD2 as novel potential targets of human NAA40 based on their N-terminal sequence. Subsequently, in vitro enzymatic assays showed that peptides representing the N-terminal sequence of these proteins can be acetylated by NAA40. Next, focusing on histone variant H2A.X, we validated the N-terminally acetylated form of this histone (Nt-acH2A.X) intracellularly using mass-spectrometry, and additionally showed that NAA40 specifically interacts and is able to N-terminally acetylate this histone variant*.* Furthermore, we provide evidence that Nt-acH2A.X is responsive to UV-induced DNA damage and its associated enzyme NAA40 affects cell survival upon UV exposure, suggesting a possible role of this modification in DNA damage response. Collectively, these findings reveal H2A.X as a *bona fide* substrate of NAA40, opening new avenues for investigating the role of this novel histone mark, as well as well as exploring the other identified potential targets of NAA40.

## Methods

### Cell culture

The HCT116 colorectal cancer cell line was kindly provided by Dr. Pantelis Hatzis (Biomedical Sciences Research Center ‘Alexander Fleming’). Cells were cultured in McCoy’s 5a medium (Gibco, Invitrogen) supplemented with 10% fetal bovine serum (FBS) (Gibco, Invitrogen). MDA-MB-231 cells (ATCC) were cultured in 90% DMEM w/stable Glutamine + 10% FBS (North America origin). SUM159T cells (ASTERAND) were grown in SUM159PT (ASTERAND) 95% Hams'F12 supplemented with 5% FBS (south America origin), 2 mM l-Glutamine, 5 μg/mL Insulin, 1 μg/mL Hydrocortisone and 10 mM HEPES. MCF10A and MCF12 cells (ATCC) were cultured in 95% DMEM:Ham's F12K (1:1) supplemented with 5% Horse serum, 2 mM L-Glutamine, 20 ng/mL hEGF, 100 ng/mL Cholera Toxin, 10 μg/mL insulin and 0.5 μg/mL Hydrocortisone. All the media were supplemented with 1% penicillin/streptomycin (Gibco, Invitrogen). Cells were grown in a humidified atmosphere at 37 °C containing 5% CO2 and were routinely tested for mycoplasma contamination. The Colorectal Cancer (CRC) cell line was used to construct dox-inducible shRNA-knockdown lines for NAA40 or Scramble (SCR) control and dox-inducible shNAA40 that ectopically express either a wild type NAA40-V5 or a pLenti/V5-empty vector (Empty Vector), as previously described [9, 21].

### UVB irradiation

Cells were irradiated in an Opsytec Dr. Grobel BS‐02 irradiation chamber. Only Ultraviolet B (UVB) irradiation (λ = 280–315 nm) was applied in this study. The radiation dose was measured with calibrated sensors and controlled using a UV‐MAT radiation controller.

### Data analysis

To characterize the presence and evolutionary conservation of the NAA40 recognition motif, the 2021-Q3 UniProt dataset containing sequences from 565254 reviewed proteins was obtained. These proteins were then reviewed for the presence of an N-terminal “MSGRG” or “SGRG” sequence. Sequence alignments were performed with Clustal omega. Default settings of the SEEK webtool were used to rank all genes according to their co-expression with NAA40 across 1652 cancer datasets. CCLE RNA-Seq data were downloaded from the DepMap portal (https://depmap.org/portal/achilles/) and TCGA data from UCSC Xena (https://xena.ucsc.edu/).

### Protein co-immunoprecipitation

Ten million cells were harvested in 1X PBS and lysed in Buffer S (10 mM HEPES, 10 mM KCL, 1.5 mM MgCl_2_, 0.34 mM sucrose, 10% glycerol) plus 0.1% Triton X-100 and 1X protease inhibitor cocktail) on ice for 10 min. Following centrifugation at 1300*g* for 5 min at 4 °C, the supernatant S1 was centrifuged at maximum speed for 10 min and the supernatant S2 was taken as the cytoplasmic fraction. Pellet was washed with 5 × pellet volume Buffer A (10 mM, 1.5 mM MgCl2, 10 mM KCL) plus 100 mM NaCl and 1 mM DTT followed by centrifuge at 3000*g* for 5 min at 4 °C. In order to purify the nuclear fractions, pellets were then re-suspended in a low-salt lysis buffer (20 Mm Hepes, 1,5 mM MgCl_2_, 150 mM NaCl, 0,2 mM EDTA, 25% glycerol) plus 0.1% v/v NP-40, 0,2 mM DTT and protease inhibitors (0.5 mM PMSF and 1X protease inhibitor cocktail) and treated with 200 units/mL of benzonase (Sigma E1014) for 15 min at 37 °C. Following sonication for 2 × 5 s pulses with a Bioruptor (Diagenode) cells were centrifuged at 16,000*g* for 10 min at 4 °C to obtain the supernatant that represents the low- salt nuclear fractions. 50 μl of each sample was saved as input to serve as positive control. Lysates where then mixed with 25 μl of Protein A Dynabeads (Thermo Fischer Scientific, 10001D) that were pre-incubated with 3 μg of V5 antibody (Sigma-Aldrich, AB3792) for 1 h at RT. Following 4 h incubation with constant agitation at 4 °C, the antibody-beads-protein complexes were centrifuged and washed three times with washing buffer (50 mM Tris–HCL pH7.5, 1 mM EDTA, 150 mM NaCl, 0.05% NP-40) and IP samples were eluted in 2X Laemmli buffer at 95 °C for 10 min.

### Immunoblotting

Samples were boiled in Laemmli buffer and equal volumes for Inputs and immunoprecipitates were loaded. Proteins were resolved by SDS-PAGE with size markers (Thermo Fisher Scientific, PI26620) and transferred to nitrocellulose membranes (GE Healthcare). After blocking with 3% TBS-T/BSA for 1 h at RT, the membranes were incubated with the primary antibodies overnight at 4 °C. The antibodies we used in this study were: V5 (Sigma-Aldrich, AB3792), NAA40 (Abcam Ab106408), H2A.X (Abcam ab11175), H2A (Abcam 18255), H4 (Merch 05–858) and PCNA (Abcam Ab29), ACTIN (Abcam Ab8227). For secondary antibody a Horseradish peroxide (HRP)-conjugated goat anti-rabbit IgG (Thermo Fisher Scientific) was used at a dilution of 1:30,000 and an HRP-conjugated goat-anti mouse IgG (P0447, Dako) was used at a dilution of 1:1000.

### RNA extraction and qRT-PCR

Total RNA was extracted using the RNeasy Mini kit (Qiagen) according to the manufacturer’s instructions. An amount of 0.5 μg total RNA was then reverse transcribed to complementary DNA using the PrimeScript RT reagent kit (Takara) with random primers. qRT-PCR was carried out using KAPA SYBR Green (SYBR Green Fast qPCR Master Mix) and the Biorad CFX96 Real-Time System. Expression data were normalized to the mRNA levels of the β-actin housekeeping gene and calculated using the 2 − ΔΔCt method. Primer sequences were obtained from IDT: *NAA40* F- TGGTGCCTACCAGTTCTTCA, R-CTCCGGCTCAGGATCTCATA, *β-actin* F-GGCATCCTCACCCTGAAGTA, R-AGGTGTGGTGCCACATTTTC, *RNA18S* F-CCCGTTGAACCCCATTCGTGA. R-GCCTCACTAAACCATCCAATCGG.

### hNAA40 recombinant protein expression

hNAA40 (NCBI: NM_024771.4) WT and E139Q were cloned in pGEX-5 × 1 vector. The plasmids were transformed in BL21 (DE3) codon plus RIL pACYC E. coli cells, generously provided by Till Bartke (Institute of Functional Epigenetics, Helmholtz Munich). The transformed bacteria were grown in 40 mL Lysogeny Broth (LB) medium complemented with 100 μg/mL Ampicillin (Sigma A9518) and 25 μg/mL Chloramphenicol (Sigma C0378-5G) for 16 h at 37 °C. Final culture volume was adjusted to 400 mL with LB and the growth was resumed for 2 h. Protein expression was initiated by adding 0.1 mM isopropyl β-D-1-thiogalactopyranoside (IPTG) (Thermo Fisher Scientific 15529019) and incubating further for 4 h at 37 °C. Bacteria were pelleted and resuspended in ice cold PBS/1% Triton-X100 supplemented with protease inhibitors. Cells were lysed by small probe sonication (amplitude 30%, 3 × 45 s on ice). Lysate bacteria were pelleted, and the supernatant was incubated with Glutathione agarose beads (Cytiva 17075601) for 1 h. Then, washing steps were made with ice cold PBS/1% Triton-X100 and ice-cold PBS. The beads with the recombinant GST-NAA40 WT or E139Q were stored at − 20 °C in 80% PBS/20% glycerol. To obtain the GST tagged hNAA40, the buffer 50 mM Tris HCL pH 8, 20 mM reduced Glutathione-20% glycerol was used to elute the GST-NAA40. To obtain the untagged version of hNAA40, hNAA40 was enzymatically cut by Factor Xa (NEB P8010S) from the beads and was dialyzed in 50 mM Tris–HCL pH 7.5, 100 mM NaCl, 25% Glycerol. The recombinant proteins were confirmed by SDS-PAGE and the concentrations were determined using the BIO-RAD protein assay dye reagent (BIO-RAD 5000006) and Nanodrop 1000.

### Fluorescence-based in vitro enzymatic assay using the recombinant hNAA40

An adaptation of the previous described fluorescence assay [22] was used to study the kinetics of hNAA40 with the histone substrates H2A.X, H2A, and H4. Specifically, the full-length histone proteins H2A.X (TEBU BIO 23615-0307), H2A, H4, and H2B, and 17-mer peptides corresponding to the N-terminal end of H4, H2A.X, H2A.J, SMARCD2 were tested with WT or E139Q hNAA40. The peptides used in this study were synthesized by BIOSYNTAN GmbH, with > 95% purity. Final substrate concentration was 15μM for the rate comparison between WT and E139Q hNAA40 (ΔRFU/min), and for the K_m_ determination the concentration ranged from 0.1 μM to 25 μM (1:2 dilutions). The reactions were in triplicate, repeated two times and contained 15 μM Thioglo4 (Merck 595504), 10 μM Acetyl coenzyme A lithium salt (Santa Cruz sc-214465), 25 mM HEPES pH 7.5, 150 mM NaCl, 0.01% Triton X100), 0.1μM hNAA40 in a final volume of 40 μl. The reaction was initiated with the addition of the substrate. The fluorescence was monitored with Tecan Spark multimode reader with excitation 410 ± 20 nm and emission 475 ± 20 nm, and the duration of the reaction was 5 min. We generated the standard curve y = 685.2*X-92.53 using 0–10 μM 1:2 dilutions of Coenzyme A sodium salt hydrate (Merck C13144) and 25 mM HEPES pH 7.5, 150 mM NaCl, 0.01% Triton X100. GraphPad Prism 8 was used for the enzyme kinetics analyses.

### MS-based analysis of histone PTMs.

Histones were enriched from HCT116 cells as previous described [23], “medium input protocol”). To analyze histone H2A.X N-terminal acetylation, approximately 2 μg of histone octamer were digested with the LysC protease (Promega), following the manufacturer’s guidelines. Peptide were separated by reversed-phase chromatography on an EASY-Spray column (Thermo Fisher Scientific), 25-cm long (inner diameter 75 µm, PepMap C18, 2 µm particles), which was connected online to a Q Exactive Plus instrument (Thermo Fisher Scientific) through an EASY-Spray™ Ion Source (Thermo Fisher Scientific). Solvent A was 0.1% formic acid (FA) in ddH2O and solvent B was 80% ACN plus 0.1% FA. LysC-digested samples were separated with a 60 min 0%–40% gradient of solvent B, at a flow rate of 300 nL/min. The Q Exactive Plus instrument was operated in the data-dependent acquisition (DDA) mode to automatically switch between full scan MS and MS/MS acquisition. Survey full scan MS spectra (m/z 375–1650) were analyzed in the Orbitrap detector with a resolution of 70,000 at m/z 200. The 12 most intense peptide ions with charge states comprised between 2 and 4 were sequentially isolated to a target value for MS1 of 3 × 106 and fragmented by HCD with a normalized collision energy setting of 28%. The maximum allowed ion accumulation times were 20 ms for full scans and 45 ms for MS/MS, and the target value for MS/MS was set to 1 × 10^5^. The dynamic exclusion time was set to 20 s, and the standard mass spectrometric parameters for all experiments were as follows: spray voltage of 1.8 kV, no sheath and auxiliary gas flow.

The acquired raw data were analyzed using the integrated MaxQuant software v.1.6.2.3 (Max Planck Institute of Biochemistry, 10.1038/nbt.1511). The UniProt HUMAN_histones 1502 database was used for histone peptide identification. Enzyme specificity was set to LysC. The estimated false discovery rate (FDR) of all peptide identifications was set at a maximum of 1%. Two missed cleavages were allowed, and the minimum peptide length was set to 6 amino acids. Variable modifications included lysine acetylation and protein N-terminal acetylation (+ 42.010 Da). Identifications and retention times were used to guide the manual quantification of peptides using QualBrowser version 2.0.7 (Thermo Fisher Scientific). The site assignment was evaluated using QualBrowser and MaxQuant Viewer from MS2 spectra. Extracted ion chromatograms (XICs) were constructed for each doubly charged precursor, based on its m/z value, using a mass tolerance of 20 ppm and a mass precision up to four decimals. The area under the curve (AUC) for the (N-ac)SGRGK(ac)TGGK peptide was normalized on histone H2A.X unmodified peptides (ATQASQEY and/or KATQASQEY).

In parallel, approximately 4 μg of histone octamer were mixed with an equal amount of heavy-isotope labelled histones, which were used as an internal standard (super-SILAC mix, [24] and separated on a 17% SDS-PAGE gel. Histone bands were then digested with the PRO-PIC protocol and acquired as described [25]. PRO-PIC digested samples were processed using the EpiProfile 2.0 software [26] and histone H3, H4 and H2A methylations and acetylations were analysed as described [25]. The AUCs for the unmodified histone H2A.X peptides KGHYAER and GKTGGKAR, and H2A.XK5ac were divided by the AUCs of the heavy forms of the same peptides in the super-SILAC spike-in. Unmodified H2A.X peptides were normalized to the levels of histone H2A. All the data were further normalized to the average value of control (WT) samples. The mass spectrometry proteomics data have been deposited to the ProteomeXchange Consortium [27], via the PRIDE partner repository with the dataset identifier PXD050981.

### Peptide pull-down

Dynabeads MyOne Streptavidin T1 (Invitrogen 65601) were used with biotinylated peptide substrates H4 (1–17), Ac-H4 (1–17), H2A.X (1–17), Ac-H2A.X (1–17), negative control peptide. The binding buffer contained 15mM NaCl, 50mM Tris–HCL pH 8, 1mM EDTA, 1% NP40 and the subsequent washing steps contained 300mM NaCl, 50mM Tris–HCL pH 8, 1mM EDTA, 2% NP40. Anti-GST (HFR) antibody (Merck A7340) was used for protein detection. For peptide loading control we eluted the peptides in 0.1X Laemmli without Bromophenol Blue) incubated for 5 min at 120 °C, vortex, and 5 min on ice, for a total of 5 cycles. Dot Blot was used for peptide detection using anti-Biotin antibody (Santa Cruz sc-101339).

### Clonogenic survival assay

HCT116 shScramble (SCR) and shNAA40 cells were treated with 1 μg/mL doxycycline for 96 h. Cells were then seeded into 6 well plates in triplicates at a density of 1000 cells/well. UVB irradiation was applied in an Opsytec Dr. Grobel BS‐02 irradiation chamber, then the cells were allowed to grow for 10–14 days. The cells were fixed with a 4% PFA for 10 min at room temperature, followed by staining with 0.5% crystal violet (Sigma-Aldrich, C6158) diluted in methanol. Following incubation for 20 min with gentle agitation at RT, cells were washed 5 times with water and air dried overnight. Sorensen buffer (0.1M sodium citrate, 50% ethanol) was used to extract the stain, and the absorbance was read at 570 nm by using a Perkin Elmer Wallac Victor 1420–002 Multilabel Counter.

### Statistical analysis

Statistical analysis was carried out using GraphPad Prism (v.6.01, La Jolla, CA). All presented data are the mean ± s.d. of at least three independent experiments and comparisons between groups were performed using Unpaired Student’s t test unless otherwise stated in the figure legend. Differences with *p < 0.05 were considered to be statistically significant.

## Results

### Presence and evolutionary conservation of the NAA40-targeting motif ‘SGRG’ in the human proteome

**Fig. 1:**
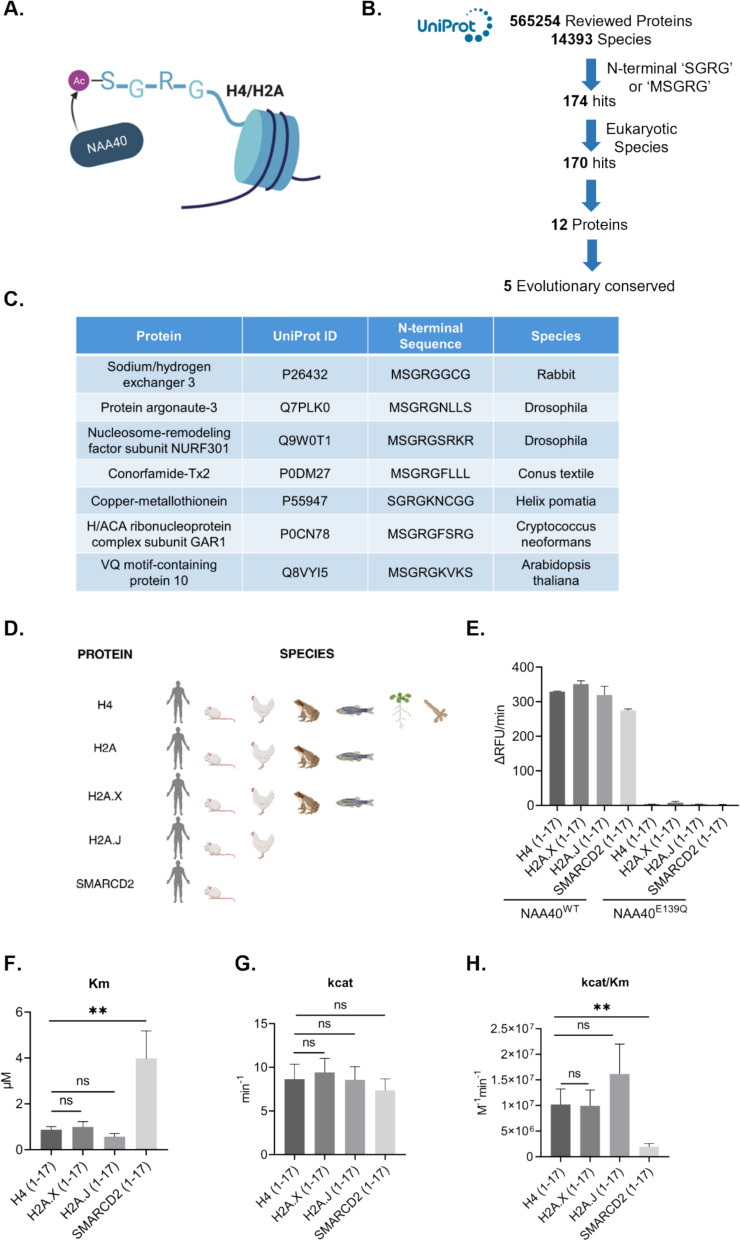
Identification of potential novel human NAA40 substrates. **A** Human NAA40 is known to acetylate the N-terminal end of proteins starting with ‘SGRG’, occurring on its two reported substrates H2A and H4; **B** Computational screening for potential NAA40 substrates based on the presence of N-terminal SGRG/MSGRG sequence; **C** Proteins containing N-terminal SGRG/MSGRG motif found in single species; **D** Schematic presenting the evolutionary conservation of the ‘SGRG’ motif of H4, H2A, H2A.X, H2A.J, and SMARCD2 across various species; **E** Quantification of Nt-acetylation of H4, H2A.X, H2A.J and SMARCD2 peptides by recombinant human wild type NAA40 (NAA40^WT^) or catalytically inactive NAA40 (NAA40^E139Q^) using a Fluorescence assay. **F** Michaelis constant (Km) and **G** Catalytic constant (kcat) values of NAA40 against H4, H2A.X, H2A.J and SMARCD2 peptides. **H** Catalytic efficiency (kcat/Km) of NAA40 against H4, H2A.X, H2A.J and SMARCD2 peptides. Statistical analyses were performed using unpaired two-tailed Student’s t test (*p < 0.05, **p < 0.01). Images created with BioRender.com

Human NAA40 is a highly specific NAT, acetylating substrates harboring an N-terminal ‘SGRG’ recognition motif (Fig. 1A; [4, 5]). To identify potential novel NAA40 substrates beyond the known H4 and H2A substrates, we screened the UniProt database composed of manually reviewed, high-confidence proteins (https://www.uniprot.org/). At the time of access (September 2021) this database contained more than half a million protein entries, collected from 14,393 species. Since the ‘SGRG’ motif is exposed after cleavage of the initiator methionine, we examined proteins for the N-terminal sequence of'SGRG'or'MSGRG'N-terminal sequence. Our analysis identified 170 hits in eukaryotic species, corresponding to 12 different proteins (Fig. 1B). For seven of these proteins a single-species hit was identified (Fig. 1C), while positive hits across a large number of species, including human, were identified for five proteins: canonical H2A and H4, H2A.X and H2A.J variants of H2A, and chromatin remodeler SMARCD2 (Fig. 1D). To ensure up-to-date findings, we repeated our analysis in December 2024. Despite an increase in the number of reviewed protein sequences, the results remained unchanged.

The H4 and H2A proteins are two of the four core histones that are part of nucleosomes, and therefore their sequences and functions are highly conserved across eukaryotic cells. For canonical H4 the N-terminal SGRG sequence was found in yeast, plant, and animal species, thus demonstrating the widest evolutionary conservation. For H2A the N-terminal SGRG sequence was found in a wide range of vertebrate and invertebrate animals (e.g. human, mouse, zebrafish, fruit fly, nematodes, and sea urchins), but not in plants or yeast (Fig. 1D).

Apart from H2A.X and H2A.J, the ‘SGRG’ motif was not present in the N-terminus of any other H2A or H4 variants (H2A.Z, H2A.W, H2A.P, H2A.B, macroH2A1, macroH2A2, or H4G) (Suppl. Fig. S1). Notably, H4G, as well as macroH2A1 and macroH2A2 variants, differ from the consensus N-terminal motif by just a single amino acid (Suppl. Fig. S1). However, even such minor variations from the ‘SGRG’ sequence have been shown to markedly reduce NAA40 ability to recognize and modify target sequences [5], highlighting the enzyme’s stringent specificity. For H2A.X the N-terminal ‘SGRG’ sequence was conserved in vertebrate animals but not in unicellular eukaryotes, where canonical H2A fulfils the function of the H2A.X variant (Fig. 1D) [28, 29]. In *Drosophila*, H2A.V is the functional equivalent to H2A.X and H2A.Z together [30], but the N-terminal sequence of this variant is ‘AGGK’ (P08985). H2A.J is a recently evolved H2A variant with only five overall amino acid residue substitutions, not involving the N-terminal end [31]. Not surprisingly then, the ‘SGRG’ motif found in H2A was also conserved in H2A.J in mammalian species. SMARCD2 stood out as the only non-histone protein with the NAA40 recognition motif. For SMARCD2 the NAA40 recognition sequence was conserved across mammalian species but was not present in other SMARCD family members (Fig. 1D). In conclusion, our analysis of the proteome here shows that ‘SGRG’ N-terminal sequences are rare, a finding that aligns with previous reported observations [4]. Overall, we identified the human histone H2A variants H2A.X and H2A.J, and the chromatin remodeler SMARCD2 as possessing an evolutionary conserved NAA40-targeted motif, highlighting them as potential novel substrates of this NAT.

### H2A.X, H2A.J and SMARCD2 are potential substrates of NAA40

To experimentally determine whether NAA40 can N-terminally acetylate the identified putative targets, we employed a modified version of a previously established fluorescence based in vitro Nt-acetylation assay ([22] and see methods for details). We first validated our assay by demonstrating the ability of purified recombinant human NAA40 to Nt-acetylate a peptide corresponding to the first 17 N-terminal residues of its known target H4 (Fig. 1E). We chose this length of peptide because it was previously demonstrated that NAA40 activity on such peptide length is similar to its full-length protein substrate [5]. Notably, a catalytically inactive form of NAA40- carrying a mutation (E139Q) in its acetyltransferase domain rendering it inactive [4] did not Nt-acetylate this H4 peptide, as expected (Fig. 1E). We next tested 17 amino acid long peptides corresponding to the N-terminal end of H2A.X, H2A.J, and SMARCD2 in our in vitro assay. Similar to what was observed for H4, these peptides were Nt-acetylated by the wild-type, but not the catalytically inactive human NAA40 (Fig. 1E). Subsequently, we proceeded to compare the catalytic efficiency of NAA40 for the putative substrates H2A.X, H2A.J and SMARCD2 against that of its known target histone H4. For this reason, we sought to quantify the Michaelis constant (Km) of NAA40, an inverse measure of affinity to the substrate. The analysis revealed similar affinity for the three histone peptides—H4, H2A.X and H2A.J—but significantly lower for SMARCD2, probably due to the fifth amino acid residue which in the three histone proteins H4, H2A.X and H2A.J is a lysine (K) but in SMARCD2 is an alanine (A) (Fig. 1F). Additionally, we measured the catalytic constant (Kcat) of NAA40 which represents the maximum number of substrate molecules converted to product per enzyme molecule per unit time when fully saturated. For all the examined peptides, NAA40 showed comparable catalytic constants (Fig. 1G). WT NAA40 showed similar kcat/Km values for the three histone proteins, indicating comparable catalytic efficiency (Fig. 1H). However, the catalytic efficiency of NAA40 towards SMARCD2 was significantly lower than histones, due to the observed differences in the affinity of NAA40 for the examined peptides (Fig. 1H). In agreement with this, it was previously shown that NAA40 exhibits reduced catalytic efficiency towards ‘SGRGA’ compared to ‘SGRGK’-containing peptides [5]. Altogether, our assay demonstrates the ability of NAA40 to catalyze the Nt-Ac of the three putative novel substrates in vitro.

**Fig. 2:**
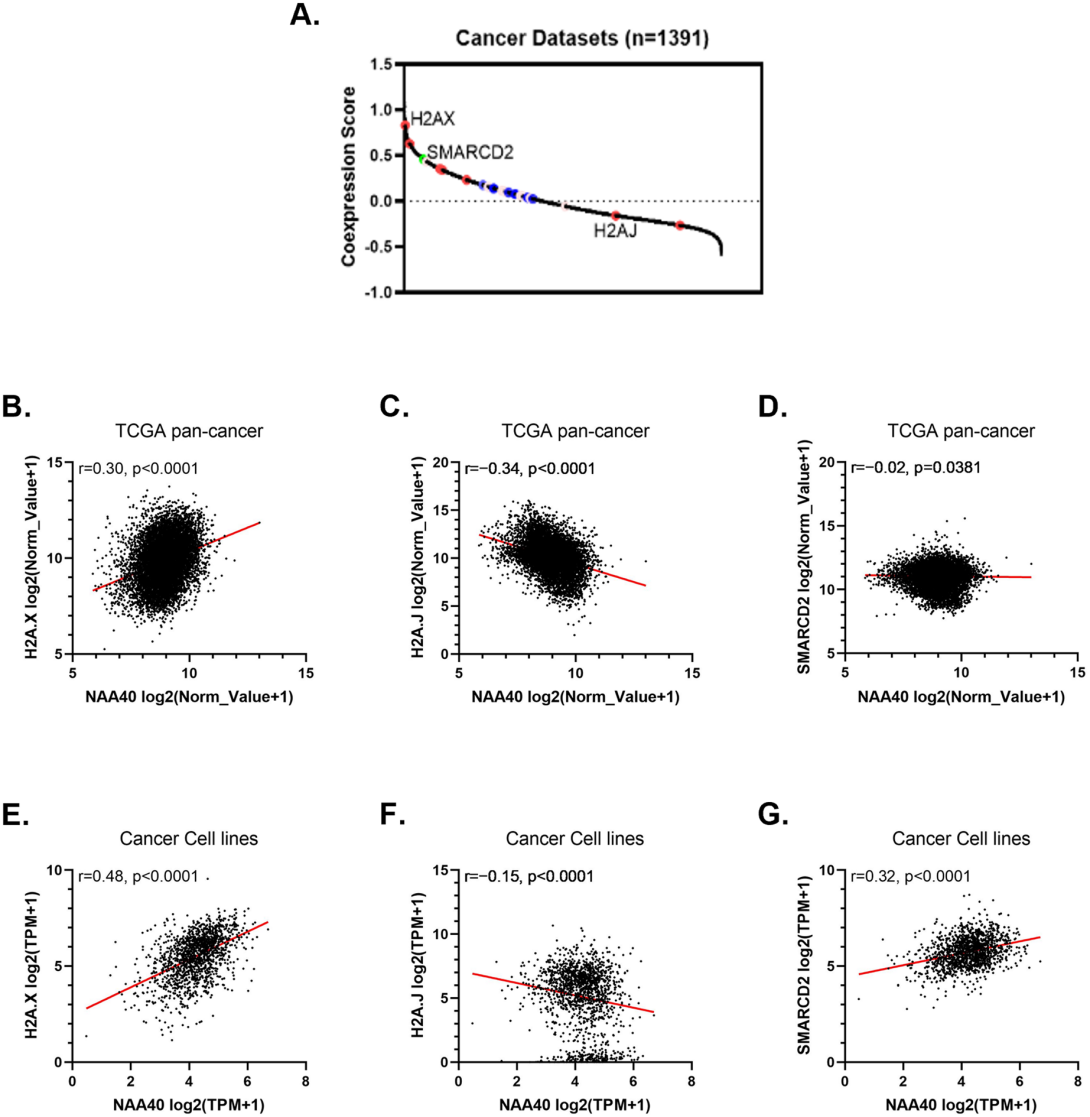
Highly correlated expression of NAA40 with H2A.X. **A** Plot depicting co-expression score between NAA40 and 17,759 human genes across 1391 datasets relating to cancer. Red circles indicate H2A variants, blue H2A isoforms, pink H4 isoforms, and the green symbol SMARCD2; **B** Co-expression measured by Pearson correlation for NAA40-H2A.X (left panel); **C** NAA40-H2A.J (middle panel) and **D** NAA40-SMARCD2 (right panel) across all samples in the TCGA dataset; **E** Co-expression measured by Pearson correlation for NAA40-H2AX (left panel); **F** NAA40-H2A.J (middle panel) and **G** NAA40-SMARCD2 (right panel) across DepMap cancer cell line panel

We then sought to determine the co-expression of NAA40 with its candidate targets because co-expression of genes could indicate their involvement in common biological pathways [32]. Hence, we used SEEK (search-based exploration of expression compendia; http://seek.princeton.edu/) to analyze the co-expression across 1652 cancer datasets of NAA40 with its putative targets. Interestingly, H2A.X was by far the most strongly co-expressed gene with NAA40 (30th out of 17,759 genes) among all known H2A-H4 isoforms and variants, as well as SMARCD2 (Fig. 2A). We next focused on the cancer genome atlas (TCGA) dataset consisting of 33 cancer types. NAA40-H2A.X displayed significant positive correlation when examining all samples together (Fig. 2B–D). Finally, the strong correlation between H2A.X and NAA40 was also replicated in the independent dataset (Cancer Cell line Encyclopedia [CCLE]) of > 1000 cancer cell lines (Fig. 2E–G). Multiple independent datasets thus concur on a transcriptional co-expression association of the two genes, H2A.X and NAA40 in cancers. In conclusion, in transcriptome profile datasets we find evidence that NAA40 and its putative target H2A.X are co-expressed and potentially coregulated.

### NAA40 physically interacts and modifies H2A.X in vitro

**Fig. 3:**
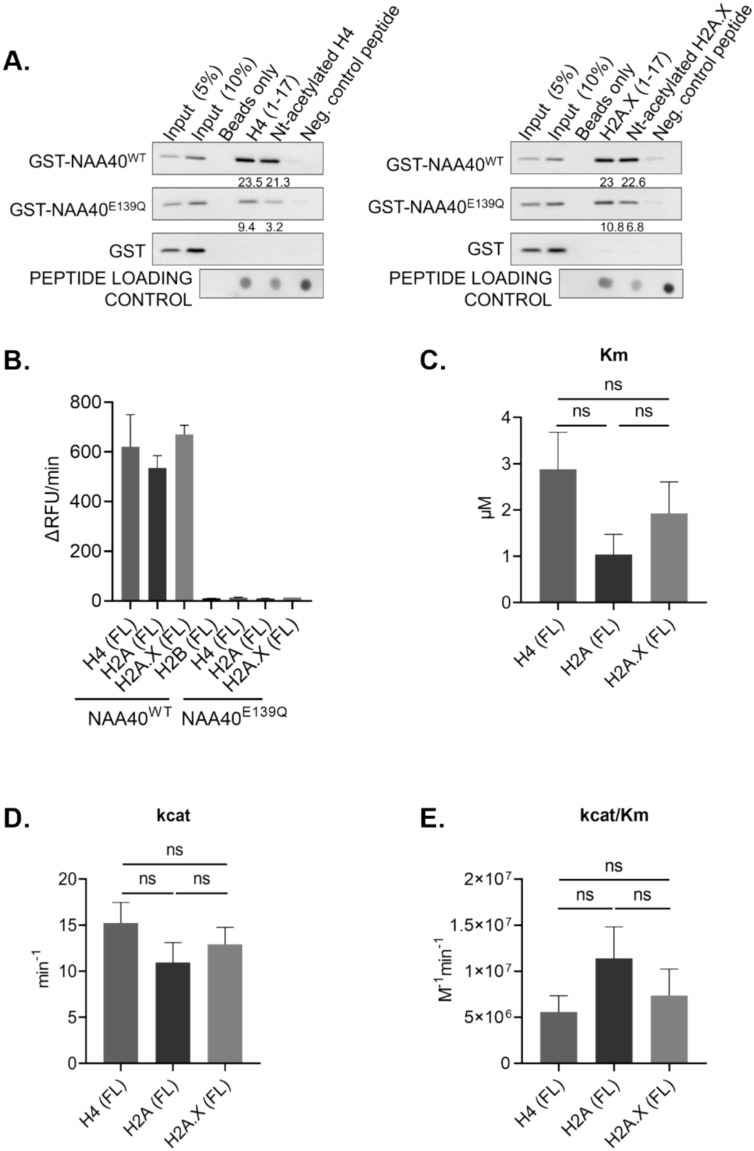
NAA40 interacts with and modifies H2A.X in vitro. **A** Peptide pull down assay investigating the direct interactions of GST-NAA40^WT^ and GST-NAA40^E139Q^ with peptides corresponding to H4, Nt-acetylated H4 (left panel), and H2A.X and Nt-acetylated H2A.X (right panel). Samples were analyzed with Western blot using GST antibody. Dot Blot was used for peptide loading detection. The numbers below each blot indicate densitometry analysis of protein levels after normalization to the peptide loading control; **B** Quantification of Nt-acetylation of full-length H4, H2A and H2A.X recombinant proteins by the wild type NAA40 (NAA40^WT^) or catalytically inactive NAA40 (NAA40^E139Q^). H2B was used as a negative control; **C** Michaelis constant (Km) and **D** Catalytic constant (kcat) values of NAA40 against H4, H2A and H2A.X recombinant proteins; **E** Catalytic efficiency (kcat/Km) of NAA40 against H4, H2A and H2A.X recombinant proteins. Statistical analyses were performed using unpaired two-tailed Student’s t test (*p < 0.05, **p < 0.01)

Motivated by the revealed co-expression of NAA40 and H2A.X, we then focused our investigation on whether histone H2A.X is a *bona fide* target of NAA40. To this end, we first examined the physical interaction between these two proteins. In order to demonstrate a direct interaction between NAA40 and H2A.X, we performed in vitro pull-down assays using peptides corresponding to either the N-terminal end of H4 as control or H2A.X, together with recombinant human NAA40. As expected, we found that unacetylated and Nt-acetylated H4 peptides bind to recombinant wild-type NAA40 (Fig. 3A, left blots). This binding occurred to a much lesser extent with the catalytically inactive form of NAA40 (Fig. 3A, left blots), indicating the importance of the enzymatic activity for mediating the interaction with the substrates, as previously suggested [4]. Notably, we detected a similar binding profile for H2A.X peptides with WT and catalytic inactive NAA40 as with H4 (Fig. 3A, right blots), supporting that H2A.X is a target of NAA40.

Subsequently we sought to determine whether the above observed binding leads to N-terminal acetylation of histone H2A.X. In pursuit of this, we performed the previously described fluorescence based in vitro Nt-acetylation assay using recombinant full-length histone proteins as substrates together with wild type GST-tagged NAA40 (WT) or the catalytically inactive form of NAA40 (E139Q). We found that WT NAA40 is able to acetylate as expected the known substrates H4 and H2A, but not the control histone H2B. Most importantly, we found that the full-length H2A.X protein is also acetylated via NAA40 (Fig. 3B). The detected activity was indeed mediated by recombinant NAA40 because the catalytically inactive form of NAA40 did not catalyze Nt-Ac for any of the examined proteins (Fig. 3B). Consistent with the histone peptides (Fig. 1E, F), WT NAA40 showed similar km, Kcat and kcat/Km values among its known targets and H2A.X, indicating comparable catalytic efficiency for the full-length proteins (Fig. 3C–E). Altogether these findings demonstrate a physical interaction between NAA40 and H2A.X, and also show that NAA40 is able to acetylate H2A.X in vitro.

### H2A.X is N-terminally acetylated within cells by NAA40

**Fig. 4:**
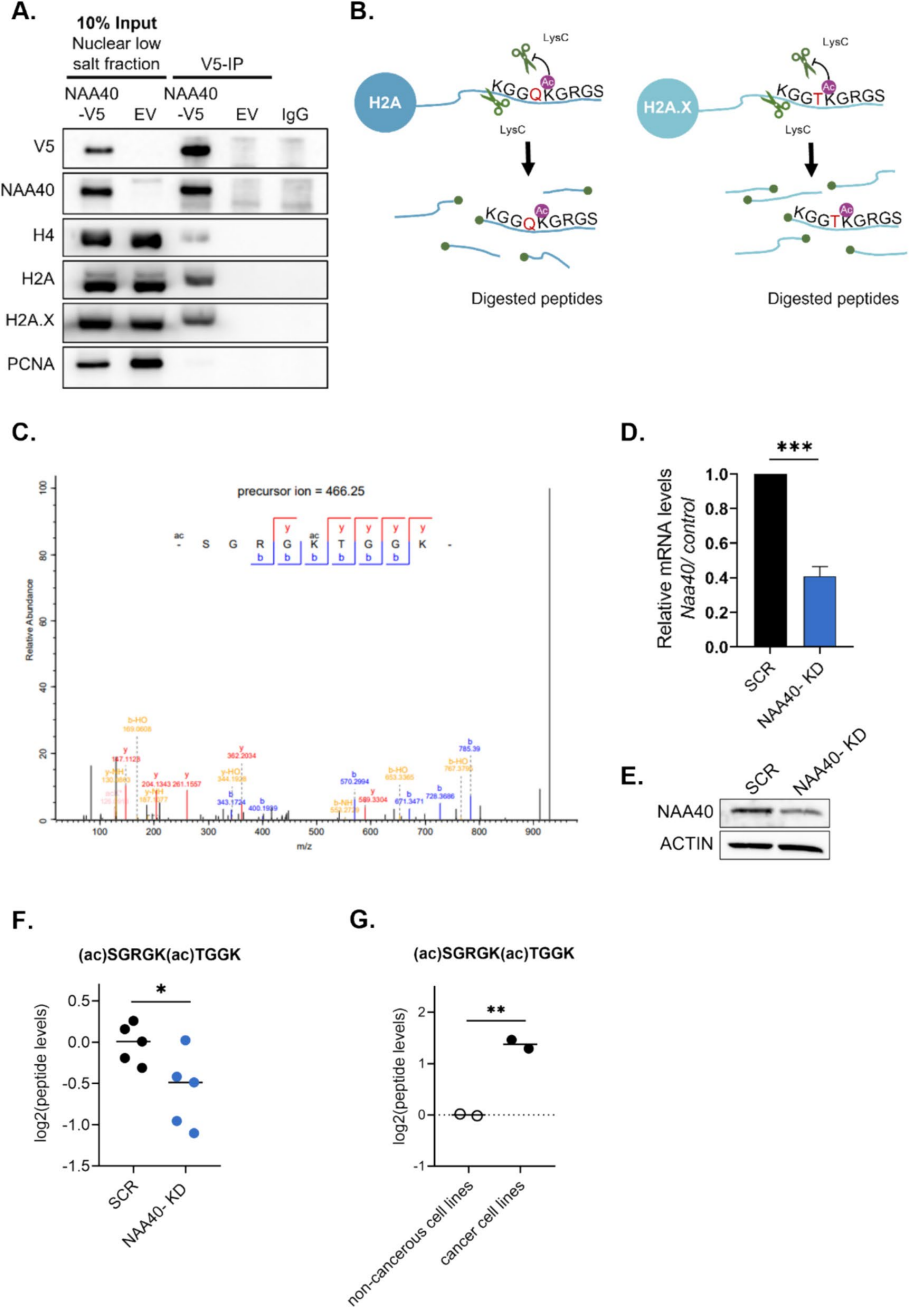
Histone H2A.X is N-terminally acetylated by NAA40 within cells. **A** Co-Immunoprecipitation of NAA40 tagged with V5 using an antibody against V5. Nuclear lysates from HCT116 cells in which the expressed NAA40 carries a V5-tag were used for IP, while empty vector (EV) cells, were used as negative control. 10% Input shows expression and loading of proteins. V5-IP shows immunoprecipitated proteins. IgG was used as an additional negative control. Samples were analyzed by Western blotting with the indicated antibodies; **B** Schematic showing the approach used in mass-spec (MS) analysis to distinguish the N-terminal peptides derived from H2A and H2A.X; **C** MS/MS spectrum of the (N-ac)SGRGK(ac)TGGK peptide corresponding to the N-terminal end of H2A.X after Lys-C digestion. Charged y (red) and b (blue) ions used to match the peptide sequence with N-terminal acetylation are annotated."b ions"were generated through fragmentation of the peptide bond from the N terminus, and"y ions"were generated through fragmentation of the peptide bond from the C terminus; **D** Quantitative real-time PCR (qRT-PCR) analysis of NAA40 mRNA levels normalized to β-actin performed in HCT116 cell line expressing doxycycline-inducible scramble shRNA (SCR), or shNAA40. The relative values of NAA40 transcripts represent the mean ± s.d. of three independent experiments; **E** Western blot analysis of cell extracts derived from dox-treated SCR or NAA40-KD HCT116 cells using antibodies against NAA40 and Actin as loading control; **F** MS analysis of (Nt-ac)SGRGK(ac)TGGK relative levels in HCT116 SCR and NAA40-KD cells. Data were normalized to the SCR control condition; **G** MS analysis of (Nt-ac)SGRGK(ac)TGGK levels in two breast cancer cell lines (MDA-MB-231 and SUM159) and two non-cancerous breast epithelial lines (MCF10A and MCF12). Statistical analyses were performed using unpaired two-tailed Student’s t test (*p < 0.05, **p < 0.01)

The above data so far suggest that NAA40 can acetylate H2A.X in vitro. Therefore, we next sought to examine this interaction and modification activity within cells. Based on previous observations showing that NAA40 is localized both in the cytoplasm and the nucleus [2, 9], we performed co-immunoprecipitation (co-IP) assay, using nuclear extracts from our previously engineered HCT116 colorectal cancer cell line model, which expresses an exogenous V5-tagged form of NAA40 [9]. As expected, we found that NAA40 interacts with its known substrates, histones H4 and H2A. Importantly, NAA40 also interacts with H2A.X intracellularly, at a similar extend to its two other known substrates (Fig. 4A). Subsequently, we aimed to determine if this modification occurs physiologically within cells by examining histones extracted from cells using mass-spectrometry (MS) [24]. While H2A and H2A.X have identical sequence in their first five residues ‘SGRGK’, they differ at their sixth residue, which is a glutamine (Q) in H2A, while in H2A.X is a threonine (T). Using the lysC protease, we detected the peptide N-(ac)SGRGK(ac)TGGK, which contains one miscleavage due to the lysine acetylation on residue 5, thus generating a peptide that is unique to H2A.X (Fig. 4B). Most importantly, we found that the first serine residue on histone H2A.X is acetylated, validating the physiological presence of this modification within cells (Fig. 4C).

Considering that NAA40 was able to interact and acetylate H2A.X in vitro (Fig. 3A and 3B), we then sought to determine whether this NAT enzyme is responsible for Nt-acH2A.X intracellularly. To achieve this, we quantified Nt-acH2A.X in HCT116 cells in which NAA40 was depleted [9, 21], Fig. 4D, E) and found significant decrease in the levels of this modification (Fig. 4F). Notably, this reduction was specific to the N-terminally acetylated form of H2A.X, as no changes were observed in the overall levels of H2A.X or H2A.XK5ac (Suppl. Fig. S2A- B). Although low levels of Nt-acH2A.X remain detectable, this is likely due to the partial reduction of NAA40 since no other enzymes have been reported to acetylate the N-terminus of H2A.X. Furthermore, since NAA40 is consistently found to be upregulated in cancer samples [10, 21, 33] we sought to assess the relative levels of Nt-acH2A.XK5ac in cancerous compared to non-cancerous cell lines. Specifically, we compared two breast cancer cell lines, MDA-MB-231 and SUM159, to two non-cancerous breast epithelial lines, MCF10A and MCF12, and found that Nt-acH2A.XK5ac levels were significantly higher in the cancerous cell lines (Fig. 4G). These findings collectively provide compelling evidence for the existence of the N-terminally acetylated form of H2A.X in cells, which appears to be upregulated in cancerous cells, and suggest that NAA40 mediates this modification.

### N-terminally acetylated H2A.X is responsive to UVB- induced DNA damage

**Fig. 5:**
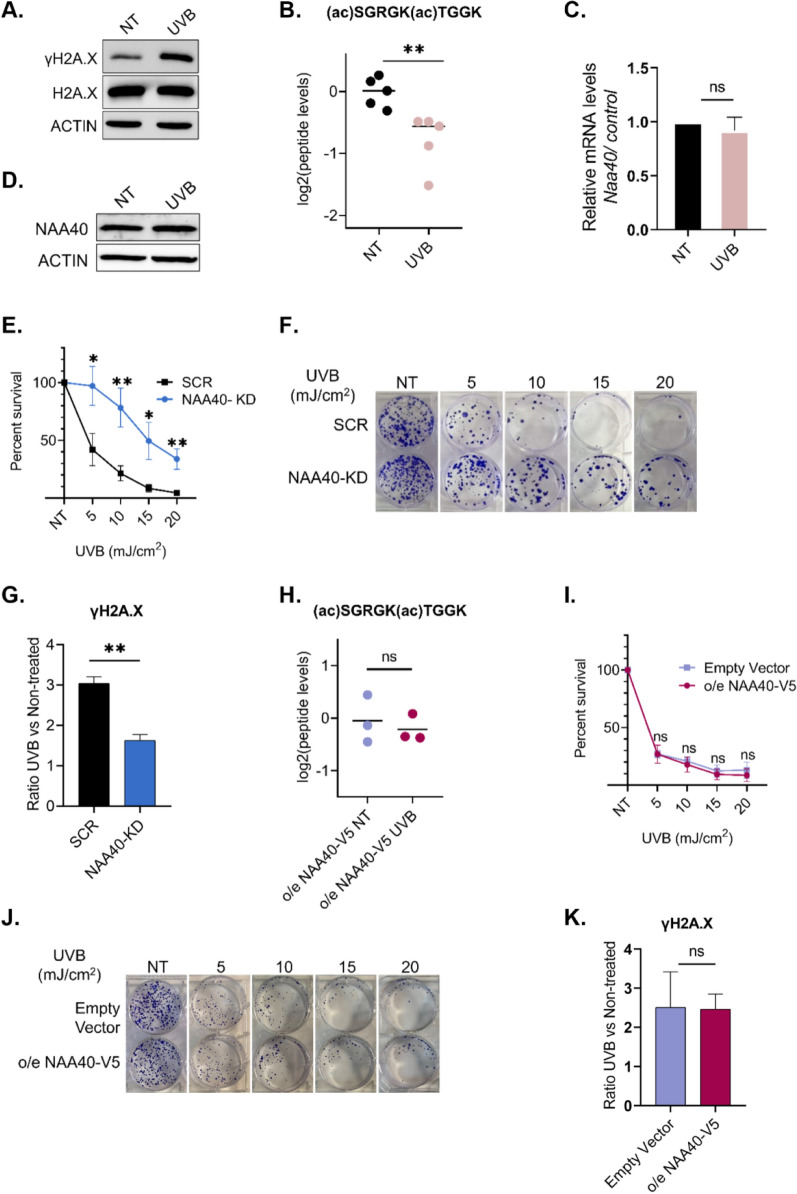
Nt-acH2A.XK5ac is reduced upon UVB irradiation and NAA40 regulates resistance to this treatment. **A** Western blot analysis of γH2A.X in non-treated (NT) or UVB-treated HCT116 cells. Cells were irradiated with UVB (10 mJ/cm^2^) and allowed to recover for 4 h. Antibodies against total histone H2A.X and Actin were used as loading controls; **B** MS quantification of (N-ac)SGRGK(ac)TGGK relative levels in HCT116 non-treated (NT) and UVB-treated cells. Data were normalized to the NT control condition; **C** qRT-PCR analysis of NAA40 mRNA normalized to *β-actin* in HCT116 NT and UVB-treated cells. Values represent mean ± SD (n = 3); **D** Western blot analysis of NAA40 in HCT116 NT and UVB-treated cells. Actin served as loading control; **E** Quantification of colony formation assay in HCT116 SCR and NAA40-KD cells NT or treated with different UVB doses. Survival rates were normalized to untreated cells. Data represent the mean ± SD (n = 3); **F** Representative images of colony formation assay in HCT116 SCR and NAA40-KD cells, either NT or treated with different UVB doses; **G** MS analysis of γH2A.X relative levels in HCT116 SCR and NAA40-KD non-treated and UVB-treated cells. Results appear as a ratio of UVB versus non-treated cells; **H** MS quantification of (N-ac)SGRGK(ac)TGGK relative levels in NAA40-V5 overexpressing (OE) non-treated and UVB-treated cells. Data were normalized to the NT control condition; **I** Quantification of colony formation assay in HCT116 NAA40-V5 OE and EV cells NT or treated with different UVB doses. Survival rates were normalized to untreated cells. Data represent the mean ± SD (n = 3); **J** Representative images of colony formation assay in HCT116 NAA40-V5 OE and EV cells, either NT or treated with different UVB doses; **K** MS analysis of γH2A.X relative levels in HCT116 NAA40-V5 OE and EV cells non-treated and UVB-treated cells. Results appear as a ratio of UVB versus non-treated cells. Statistical analyses were performed using unpaired two-tailed Student’s t test (*p < 0.05, **p < 0.01)

Based on a previous study showing that a large number of histone acetylation sites are reduced in response to UVB-induced DNA damage [34] and considering the crucial role of H2A.X in DNA damage signaling and repair [35], we sought to investigate whether the newly identified Nt-acH2A.X is also sensitive to DNA damage. We used UVB irradiation to induce DNA damage which is signified by induction of the damage signaling modification γH2A.X (Fig. 5A). Using MS analysis [25], we first examined the acetylated forms of canonical histones and confirmed the previously reported histone-wide reduction of acetyl modifications in response to UVB- induced DNA damage (Suppl. Fig. S3 and [34]). Next, we found that upon UVB- induced DNA damage, the levels of Nt-acH2A.XK5ac are also reduced, while the total levels of H2A.X remain unchanged (Fig. 5B and Suppl. Fig. S4A). Additionally, we found that H2A.XK5ac is also decreased upon UVB irradiation (Suppl. Fig. S4B). Notably, NAA40 mRNA and protein levels remain unchanged after UVB treatment (Fig. 5C, D), indicating that the decrease in Nt-acH2A.X levels is not due to reduced NAA40 expression or abundance. These results suggest that N-terminal acetylation of H2A.X is responsive to DNA damage, similarly to other acetylated histone residues, which is proposed to be a coping mechanism to UV-induced DNA damage [34]. To explore a potential cellular effect of this modification, we examined the response of cells to UVB- induced DNA damage in the presence and absence of its associated enzyme NAA40. Clonogenic survival assays showed that NAA40-depleted cells were significantly more resistant to UVB-induced DNA damage (Fig. 5E, F), while, mass spectrometry analysis revealed reduced γH2A.X levels in these cells (Fig. 5G, Suppl. Fig. S4C). Importantly, we also examined the response in HCT116 cells overexpressing V5-tagged NAA40 and found that Nt-acH2A.XK5ac levels remained unchanged after UVB treatment (Fig. 5H, Suppl. Fig. S4D, E). Accordingly, cell overexpressing NAA40 and control cells transfected with Empty Vector (EV) showed similar sensitivity to UVB and γH2A.X levels (Fig. 5I–K, Suppl. Fig. S4F). These results suggest that NAA40 influences the DNA damage response, potentially through H2A.X N-terminal acetylation. Taken together, our findings indicate that histone H2A.X is directly N-terminally acetylated by NAA40, and implicate this modification in UV-induced DNA damage.

## Discussion

The identification of the yeast NAA40 homolog as a highly selective NAT acting specifically on histones H2A and H4 was first reported more than 20 years ago [3]. Isolation and characterisation of human NAA40 documented the same high specificity towards histones H2A and H4 [2], which was subsequently demonstrated that this selectivity depends on the first four N-terminal residues of these substrates [4, 5], composed of serine(S)-glycine(G)-arginine(R)-glycine(G). Notably, it has been shown that even minimal deviations from the consensus ‘SGRG’ sequence severely impair NAA40 recognition and activity [5]. In this study, we identify the evolutionary conserved presence of the NAA40 recognition motif (SGRG), in three additional human proteins, and experimentally verify the ability of this NAT to Nt-acetylate their N-termini by utilising a specific biochemical assay. Thus, through this study we expand the pool of potential substrates of the human NAA40 beyond the known canonical histone H2A/H4 substrates.

We based our initial in silico investigation on the presence and evolutionary conservation of the human ‘SGRG’ NAA40 recognition motif by searching the UniProt protein database. Although this analysis identified new potential NAA40 substrates, it is possible that there are still unidentified targets for two main reasons. The first possibility is that it remains likely that there are as-of-yet unidentified protein isoforms possessing this N-terminal motif, generated for example through alternative splicing or post-translational cleavage that would reveal an originally internal ‘SGRG’ sequence. Given the rarity of the N-terminal ‘SGRG’ motif among currently identified proteins, we anticipate that such unidentified proteins will be few in number, if they do actually exist. Secondly, it remains a possibility that the human NAA40 can Nt-acetylate protein substrates beyond those having the ‘SGRG’ N-terminal sequence. However, previous structural and biochemical investigations of the human NAA40 protein argue against this possibility and support a highly stringent specificity for NAA40 activity. In particular, substitutions of residues in the ‘SGRG’ sequence have been shown to significantly affect the interaction and catalytic activity of NAA40 [4, 5]. Moreover, this is supported by the fact that expression of human NAA40 in yeast cells targets only histone H4 which possesses the SGRG motif and not histone H2A which carries an SGGK N-terminal sequence [2, 8, 36]. Importantly, alternative isoforms of NAA40 have been recently identified [37, 38], but whether these isoforms exhibit differences in selectivity remains unclear. It is therefore probable that our current analysis has potentially identified the complete or near complete target repertoire of human NAA40.

The evolutionary conservation of amino acid residues can indicate residues that are important for the stability and/or function of the protein [39]. It is important to note therefore the deep evolutionary conservation of the five identified human proteins with an N-terminal ‘SGRG’ motif (Fig. 1D). In species where modest deviations from the human NAA40 recognition motif occur, it is still possible that the NAA40 orthologs from those species have distinct structure and biochemistry that allows them to Nt-Ac those proteins even without strictly possessing the ‘SGRG’ sequence. One such previously reported example is the yeast NAA40 which is able to Nt-acetylate the divergent ‘SGGK’ N-terminal sequence of histone H2A, unlike its human homolog [2]. This promiscuity of yeast NAA40 might also explain the observation that H2A.Z and Lge1 may represent new substrates even though these proteins do not contain the ‘SGRG’ motif, instead featuring SGYT for Lge1 and ‘SGKA’ for H2A.Z [37].

Motivated by the transcriptional co-expression of *NAA40* and *H2A.X*, we proceeded to demonstrate that this histone variant is a new *bona fide* target of NAA40. First, mass spectrometry analysis allowed us to experimentally demonstrate the presence of an N-terminally acetylated form of H2A.X intracellularly, which is decreased in the absence of NAA40. Second, we showed the physical interaction of the two proteins, in vitro and within biological cells. Interestingly, our results suggest that NAA40 catalytic activity may influence substrate binding stability. The E139Q mutation, which replaces the catalytically essential glutamic acid with the uncharged glutamine, results in reduced substrate binding in our peptide pull-down assay. This indicates that the negative charge of Glu139 may contribute not only to catalysis but also to electrostatic interactions that stabilize substrate binding, consistent with previous findings shown that NAA40 Glu139 forms hydrogen bonds with Ser1 [4]. Additionally, although histone N-terminal acetylation is generally considered to occur mainly in a co-translational manner [40], in the present study we showed that NAA40 physically interacts with H2A.X inside the nucleus, suggesting that H2A.X N-terminal acetylation might occur also post-translationally. Importantly, this finding aligns with recent observations from our lab, showing that the yeast ortholog Nat4 is localised on chromatin [36]. Third, using a biochemical assay we showed that NAA40 can Nt-Ac full-length recombinant H2A.X protein as well as peptides corresponding to the N-terminal sequence of H2A.X. Fourth, consistent with the reported upregulation of NAA40 in cancer [10, 21, 33], we found that Nt-acH2A.XK5ac levels were significantly higher in cancerous cell lines than in non-cancerous cells. Overall, the above evidence clearly designate H2A.X as a new substrate for NAA40. The performance of similar experimental approaches in future studies will be required to conclusively demonstrate that the other two putative NAA40 substrates, H2A.J and SMARCD2, are also biologically valid substrates of this NAT.

H2A.X is a variant of histone H2A that represents 2–25% of mammalian H2A molecules depending on the organism and cell type [19]. This variant is involved in processes that include X chromosome inactivation, stem cell biology, cellular senescence [41], and most importantly has a crucial role in the signalling of DNA damage response (DDR) [19]. The central role of H2A.X in the repair of DNA damage has been established by previous studies showing that mice deficient for H2A.X are sensitive to DNA damage and display genomic instability [42, 43]. Importantly, a number of DNA damage responsive H2A.X modifications have been shown to be instrumental in initiating the eukaryotic DNA damage response and the associated chromatin remodelling. Even though the most prominent DNA damage-related PTM is the phosphorylation of histone variant H2A.X on serine 139 (γH2A.X) [19], recent findings have unveiled many other modifications on histone H2A.X that influence and support its role in DNA damage response and repair [44–50]. Collectively, these studies have shown that the engagement of H2A.X in the DDR may not be solely governed by γH2A.X, while additionally they have suggested that H2A.X may possess multiple PTMs that contribute combinatorically to a chromatin-regulated DDR. Our data also show that Nt-acH2A.XK5ac in HCT116 cancer cells is responsive to UV-induced DNA damage (Fig. 5B), proposing a possible link to this process. We assessed if NAA40 transcript and protein levels are altered post-UVB and found no change (Fig. 5C, D, suggesting that the decrease in H2A.X histone acetylation levels are not due to reduced NAA40 abundance, but remains possible that UVB alters NAA40 activity or subcellular localisation. Although lysine-directed histone deacetylases are well characterized, an enzyme capable of removing N-terminal acetyl marks has not yet been identified; consequently, a direct deacetylation mechanism cannot be evaluated at present. Instead, our data reveal a UVB-induced global loss of histone acetylation (Suppl. Fig. S3) consistent with PA200-mediated degradation of acetylated histones [34], suggesting a general loss of acetylation which could be mediated through histone exchange. Additionally, due to technical limitations we were unable to distinguish the specific effects of UVB on Nt-acH2A.X and H2A.XK5ac, and thus cannot exclude the possibility that the observed reduction in Nt-acH2A.XK5ac results from a general loss of H2A.XK5ac or due to a distinct regulatory mechanism.

Notably, in the present study, we showed that NAA40-depleted cells were significantly more resistant to UVB treatment (Fig. 5E, F), and displayed reduced γH2A.X induction (Fig. 5G), suggesting that NAA40 is implicated in the DNA damage response, potentially through H2A.X N-terminal acetylation. In support of this, Nt-acH2A.X levels remained unaltered following UVB treatment in NAA40-overexpressing cells (Fig. 5H), which consistently remained sensitive to UVB treatment (Fig. 5I, J) and properly induced γH2A.X (Fig. 5K). Together, our findings support a functional role for NAA40 in orchestrating cellular responses to genotoxic stress. This regulation may be mediated through the newly identified NAA40 substrate, H2A.X, a central player in DNA damage response. However, we cannot exclude at this point the possible contribution of previously known NAA40 targets, histone H4 and canonical H2A, in this functional impact. Intriguingly, we have recently defined a role for yeast NAA40 in DNA damage checkpoint signalling through its activity on H4 [36].

The biological function of a modifying enzyme is ultimately transmitted through its substrates. The Nt-Ac of histones H2A and H4 has been previously reported to promote oncogenic phenotypes such as invasion, growth, and chemoresistance [9, 10, 21, 51, 52]. Based on these findings we and others have argued that NAA40 is a promising candidate target for cancer therapy [6]. The discovery of further substrates opens a window for the involvement of NAA40 in additional cellular processes, relating to these newly identified protein targets. For example, as discussed previously H2A.X is involved in DNA damage, H2A.J promotes inflammatory gene expression [31], and SMARCD2 has a role in granulopoiesis [53]. It is therefore possible that human NAA40 has additional important biological and clinical functions that are mediated by substrates beyond H2A and H4.

## Conclusion

Results presented here describe the identification of histone variants H2A.X and H2A.J as well as the chromatin remodeler SMARCD2 as new potential substrates of human NAA40. Importantly, our findings unveil histone H2A.X as a novel *bona fide* substrate of NAA40 within cells with its N-terminal acetylation shown to be responsive to UV-induced DNA damage. These observations lay the foundation for further investigation into the functional and mechanistic impact of this novel histone mark, as well as exploring other identified potential targets of NAA40.

## Supplementary Information

**Additional file1. Fig. S1.** N-terminal sequences of H2A and H4 histone variants. ‘SGRG’ motif is only present in H2A.X and H2A.J histone variants and not in any other H2A or H4 variant.

**Additional file 2. Fig. S2.** MS analysis of H2A.X and H2A.XK5ac levels in HCT116 SCR and NAA40 knockdown cells. **A** MS analysis of H2A.X relative levels based on the quantification of the unmodified GKTGGKAR and KGHYAER peptides in HCT116 SCR control and NAA40 knockdown cells; **B** MS analysis of H2A.XK5ac relative levels in HCT116 SCR and NAA40 knockdown cells. In A and B, data were normalized to the SCR control condition. Statistical analyses were performed using unpaired two-tailed Student’s t test.

**Additional file 3. Fig. S3.** MS analysis histone PTM levels in HCT116 non-treated and UVB- treated cells. Heatmap display of the log2 of L/H ratiosobtained after MS quantification for the indicated histone PTMs for HCT116 non-treated control and UVB-treated cells. The grey colour indicates peptides that were not quantified. The data were normalized to the average in the control condition. Peptides were compared by multiple t-test.

**Additional file 4. Fig. S4.** MS analysis of H2A.X and H2A.XK5ac levels in non-treatedand UVB treated HCT116 cells. **A** MS of H2A.X relative levels based on the quantification of the unmodified GKTGGKAR and KGHYAER peptides in non-treatedand treated with UVB-cells; **B** MS analysis of H2A.XK5ac relative levels in HCT116 NT and UVB-treated cells. In A and B the data were normalized to the NT control condition; **C** MS of H2A.X relative levels based on the quantification of the unmodified GKTGGKAR and KGHYAER peptides in shScrambleand NAA40 knockdownnon-treated and treated with UVB-cells. Results appear as a ratio of UVB versus non-treated cells; **D** MS of H2A.X relative levels based on the quantification of the unmodified GKTGGKAR and KGHYAER peptides in NAA40-V5 overexpressingnon-treated and treated with UVB-cells; **E** MS analysis of H2A.XK5ac relative levels in NAA40-V5 OE non-treated and treated with UVB-cells. In D and E the data were normalized to the NT control condition; **F** MS of H2A.X relative levels based on the quantification of the unmodified GKTGGKAR and KGHYAER peptides in NAA40 OE and EV non-treated and treated with UVB-cells. Results appear as a ratio of UVB versus non-treated cells. Statistical analyses were performed using unpaired two-tailed Student’s t test.

## Data Availability

The mass spectrometry proteomics data have been deposited to the ProteomeXchange Consortium via the PRIDE [54] partner repository with the dataset identifier PXD050981.
