# Supplementary figures and images for: H2A.X N-terminal acetylation is a newly identified NAA40-mediated modification that is responsive to UV irradiation
Source: Epigenetics Chromatin. 2025 Jul 16;18:46. doi: 10.1186/s13072-025-00608-3 (PMC12265263; doi:10.1186/s13072-025-00608-3)

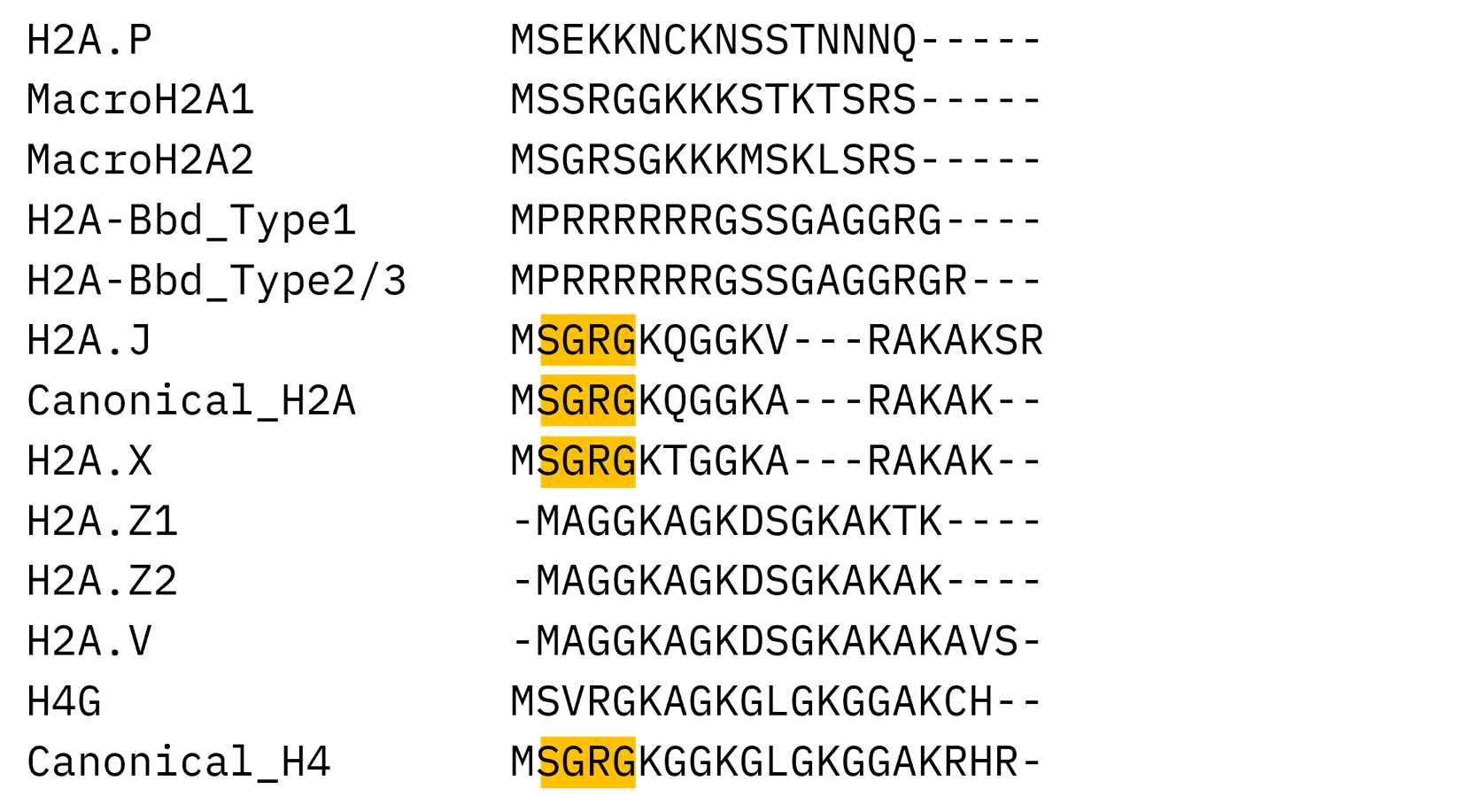

Supplement: Supplementary file 1 — Additional file1. Fig. S1. N-terminal sequences of H2A and H4 histone variants. ‘SGRG’ motif is only present in H2A.X and H2A.J histone variants and not in any other H2A or H4 variant. [file 13072_2025_608_MOESM1_ESM.tif]

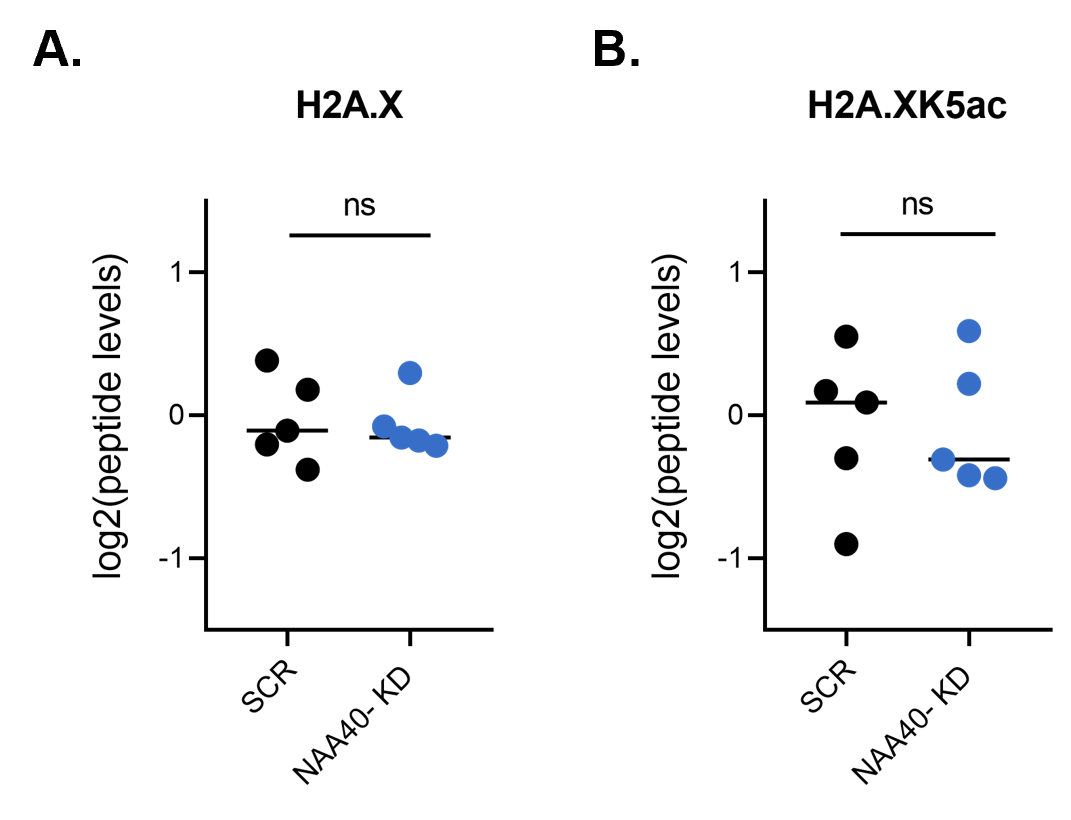

Supplement: Supplementary file 2 — Additional file 2. Fig. S2. MS analysis of H2A.X and H2A.XK5ac levels in HCT116 SCR and NAA40 knockdown cells. A MS analysis of H2A.X relative levels based on the quantification of the unmodified GKTGGKAR and KGHYAER peptides in HCT116 SCR control and NAA40 knockdown cells; B MS analysis of H2A.XK5ac relative levels in HCT116 SCR and NAA40 knockdown cells. In A and B, data were normalized to the SCR control condition. Statistical analyses were performed using unpaired two-tailed Student’s t test. [file 13072_2025_608_MOESM2_ESM.tif]

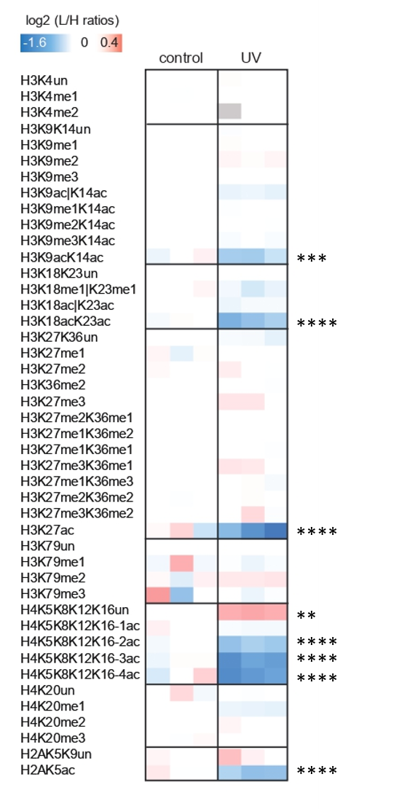

Supplement: Supplementary file 3 — Additional file 3. Fig. S3. MS analysis histone PTM levels in HCT116 non-treated and UVB- treated cells. Heatmap display of the log2 of L/H ratiosobtained after MS quantification for the indicated histone PTMs for HCT116 non-treated control and UVB-treated cells. The grey colour indicates peptides that were not quantified. The data were normalized to the average in the control condition. Peptides were compared by multiple t-test. [file 13072_2025_608_MOESM3_ESM.tif]

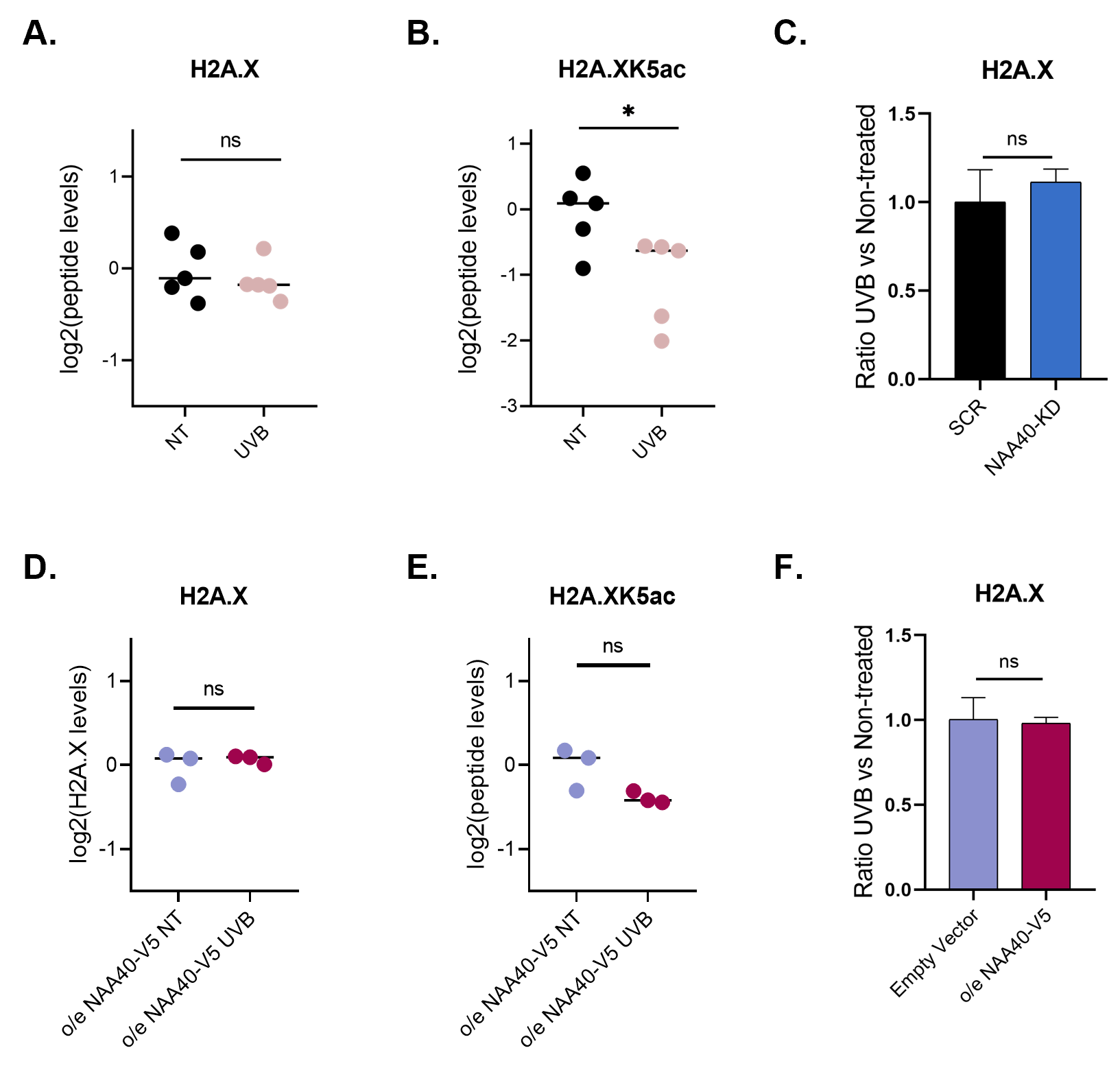

Supplement: Supplementary file 4 — Additional file 4. Fig. S4. MS analysis of H2A.X and H2A.XK5ac levels in non-treatedand UVB treated HCT116 cells. A MS of H2A.X relative levels based on the quantification of the unmodified GKTGGKAR and KGHYAER peptides in non-treatedand treated with UVB-cells; B MS analysis of H2A.XK5ac relative levels in HCT116 NT and UVB-treated cells. In A and B the data were normalized to the NT control condition; C MS of H2A.X relative levels based on the quantification of the unmodified GKTGGKAR and KGHYAER peptides in shScrambleand NAA40 knockdownnon-treated and treated with UVB-cells. Results appear as a ratio of UVB versus non-treated cells; D MS of H2A.X relative levels based on the quantification of the unmodified GKTGGKAR and KGHYAER peptides in NAA40-V5 overexpressingnon-treated and treated with UVB-cells; E MS analysis of H2A.XK5ac relative levels in NAA40-V5 OE non-treated and treated with UVB-cells. In D and E the data were normalized to the NT control condition; F MS of H2A.X relative levels based on the quantification of the unmodified GKTGGKAR and KGHYAER peptides in NAA40 OE and EV non-treated and treated with UVB-cells. Results appear as a ratio of UVB versus non-treated cells. Statistical analyses were performed using unpaired two-tailed Student’s t test. [file 13072_2025_608_MOESM4_ESM.tif]
